# Determinants of stillbirth in Bonga General and Mizan Tepi University Teaching Hospitals southwestern Ethiopia, 2016: a case–control study

**DOI:** 10.1186/s13104-017-3058-y

**Published:** 2017-12-07

**Authors:** Tensay Kahsay Welegebriel, Tegene Legese Dadi, Kebadnew Mulatu Mihrete

**Affiliations:** 1 School of Nursing, College of Health Sciences, Mekelle University, Mekelle, Ethiopia; 2 Department of Public Health, College of Health Sciences, Mizan-Tepi University, Mizan, Ethiopia; 3 School of Public Health, Department of Epidemiology and Biostatistics, College of Medicine and Health Sciences, Bahir Dar University, Bahir Dar, Ethiopia

**Keywords:** Still birth, Determinates, Mizan-Tepi, Bonga, Ethiopia

## Abstract

**Objective:**

This study aimed to identify determinants of still birth in selected hospitals of Southwestern Ethiopia.

**Result:**

A total 540 charts registered for maternal health services utilization were included in the analysis with proportion of case to control ratio of one to three (135 cases, 405 control). Women who attended antenatal care were 40% less risk for stillbirth compared to those who did not attend antenatal care (AOR = 0.6, 95% CI 0.39, 0.94). Those who had labor length ≥ 24 h were 2.4 times at risk to have still birth than ≤ 24 h (AOR = 2.44, 95% CI 1.4, 4.26). Women who developed uterine rupture were about 5 times more likely to have still birth than did not develop the complication (AOR = 4.9, 95% CI 1.67, 14.35). Women who have different antenatal risks were 4.5 times more likely to have still birth (AOR = 4.58, 95% CI 1.45, 14.48). Weight of baby ≥ 2.5 kg were 73% less likely to still birth when compared to counterparts (AOR = 0.27, 95% CI 0.14, 0.53).

## Introduction

Still birth is the death of a baby after 28 weeks of gestation based on world health organization (WHO). Around 2.65 million still births come about every single year globally, out of those 1.45 million occur during the antepartum period [1] and it is responsible for 7% of total global burden of disease [2]. The estimated stillbirth rate for developed countries was ranged from 4.2 to 6.8 per 1000 births, while, for the developing world, it ranges from 20 to 32 per 1000 births [3].

WHO survey also showed that the rate of still birth and early neonatal death was 17.7 and 8.4 million respectively across 29 countries [4]. The factors for stillbirth are also different in developed and developing world. The rate of stillbirth during labor is 1 in 1000 in developed world, whereas it is 1 per 100 births in developing world [5].

Different studies from different countries showed maternal age greater than 35 years, multiple pregnancies, parity higher than four, educational status, lack of antenatal care follow-up, chronic comorbidities, preeclampsia or APH, intrauterine growth restriction, major congenital anomaly of the infant, and poor maternal nutritional status were the most frequently mentioned factors that influences the outcome of still birth [6–8].

Prevention and cutback of stillbirth is significant in order to go in line with the current memo of the global newborn action plan by the 67 the WHO congress, aimed the global target to achieve stillbirth rate of < 10/100 births by 2035 [9].

In 2011, Ethiopia had an estimated stillbirth rate of 46 per thousand births, while in southern national nationality peoples region state (SNNPR) stillbirth rate were 29/1000 [10]. By 2030, every country should have a national a stillbirth’s rate of 12 or less per 1000 total births [17, 18]. Therefore, country planners must analyses their context-specific needs, researching the problem, and apply a rational framework for prioritizing essential services and scaling up. In order to meet the target knowing the risk factors or determinants of stillbirth is important. So it is very imperative to assess determinates of stillbirth which could play very crucial role in generating data that are important to fill gaps of limited information. Thus the objective of this research is to identify determinants of stillbirth in Bonga general and Mizan-Tepi university teaching hospitals, data taken from obstetrics and gynecology ward of the respective hospitals.

## Main text

### Methods

Institution based case control study was used to analyze the data from maternal charts registered in the logbook at obstetrics and gynecology units for different health services in Mizan-Tepi university teaching and Bonga general hospitals from January 2011–2015, SNNPR, southwest, Ethiopia. The data was extracted from December 20–January 30, 2016.

The source population were all charts of mothers registered in the hospitals for maternal health service utilization. The cases are stillbirths, which were make certain based on the status outcome obtained from the charts. Charts of mothers having live birth outcome were considered as controls. Missed charts from the archive but, registered on registration book were excluded from the study.

Sample size was determined using Epi info version 7 by taking the following assumptions: 95% CI, and 80% power, odds ratio of 1.83, case to control ratio of 1:3 and the prevalence of exposure among controls were 57% taken from unmatched case control study done in Mbarara regional hospital [11]. A total of 547 charts, 137 cases and 410 controls were included in the study. Finally, 135 cases and 405 controls charts were included in analysis the remaining was discarded due to missing information greater than 40% of the variables under study.

Records of both cases and controls were selected from, all units provided maternal health care service in the hospitals which fulfills the inclusion criteria were included in the study. Cases that are registered on the log book but whose charts’ were missed. Charts that didn’t include the status of new born (dead or alive) were excluded from the study. First cases were identified from logbook or registration book, which were found in the study period, from respective units. After identifying the cases included in the sample the time of admission was identified for each selected cases. For each case 3 controls who were survived births were selected, which were born immediately preceding or following a case.

Data extraction checklist was adapted from related surveillance. The checklist consists of relevant information on socio-demographic data, obstetric and delivery history, and birth outcome. Three midwives and three nurses were recruited and trained for 3 days as data collectors.

After data collection was completed, each completed checklist was given a unique code by the principal investigator. Data were entered using Epi Data version 3.1 then exported to STATA version 13.1 for analysis. The data were cleaned for inconsistencies and missing values. Simple frequencies were done to see the distribution of the study subject with the outcome variables and to see any missing data. Bivariate binary logistic regression was used to select variables for multivariable binary logistic regression. Multivariable binary logistic regression was used to identify determinants of still birth. Confidence interval of 95% was used to see the precision and the level of significance was taken at p value ≤ 0.05. Thus the crude odds ratio (COR) from bivariate binary logistic regression and adjusted odds ratio (AOR) from multivariable binary logistic regression is reported.

Before the data collection, ethical clearance was obtained from research and ethics committee of the college of health sciences of Mizan-Tepi University. Written permission letter was also received from the hospitals administrators. In order to be confidential only the code, not the names of the participant was taken from the chart.

### Result

A total 540 charts were included in the analysis with proportion of case to control was one to three (135 case, 405 control). The proportion of still birth with age of mother was higher (50%) in age group of greater than 34 years, 24% still birth with age of 20–34 years and 19.4% of still birth with age of less than 20 years. From all, 278 (80%) of women were attending ANC, of those who were attending ANC 69 (19%) had still birth, while the proportion of still birth from those who did not attend ANC were 66 (34.2%). Fifty (58%) of women had length of labor greater than 24 h, of which, 39 (43.8%) of their birth outcome were still birth. From those who had anemia, 4 (36.4%) of them had still birth, while, 134 (24.8%) of still birth were found from those who had not anemia. Around 17 (68%) of stillbirth was associated to uterine rupture. Thirty-two (46.4%) of still birth were related to antenatal risks like placenta previa, abruption placenta and previous cesarean section. Just about 19 (46.3%) of still birth were directly related to obstructed labor (OL) and 17 (47.2%) of still birth was contributed with different undiagnosed APH in direct or indirectly (Table 1).

**Table 1:** Obstetric characteristics of women who give birth from 2011 to 2015 at Mizan-Tepi university teaching and Bonga hospitals, 2016 (n = 540)

Variable	Cross tabulation still birth	
	No (%**)	Yes (%**)
Attending ANC*		
No	127 (65.8)	66 (34.2)
Yes	278 (80.1)	69 (19.9)
Length of labor (h)		
< 24	355 (78.7)	96 (21.3)
≥ 24	50 (58.2)	39 (43.8)
Post-term pregnancy		
No	397 (74.9)	133 (25.1)
Yes	8 (80)	2 (20)
Presence of HIV/AIDs		
No	396 (74.7)	134 (25.3)
Yes	9 (90)	1 (10)
Anemia		
No	398 (75.2)	131 (24.8)
Yes	7 (63.6)	4 (36.4)
Multiple gestation		
No	392 (75.4)	128 (24.6)
Yes	13 (65)	7 (35)
Uterine rupture		
No	397 (77.1)	118 (22.9)
Yes	8 (32)	17 (68)
Presence of co-morbidity		
No	387 (75.2)	128 (24.8)
Yes	18 (72)	7 (28)
PIH		
No	388 (75)	129 (25)
Yes	17 (73.9)	6 (26.1)
APH		
No	386 (76.6)	118 (24.4)
Yes	19 (52.8)	17 (47.2)
Antenatal risks		
No	368 (78)	103 (22)
Yes	37 (53.6)	32 (46.4)
OL		
No	383 (76.8)	116 (23.2)
Yes	22 (53.7)	19 (46.3)
Weight of the new born (kg)		
< 2.5	13 (37.1)	22 (62.9)
≥ 2.5	392 (77.6)	113 (22.4)
Age (years)		
< 20	50 (80.6)	12 (19.4)
20–34	338 (76)	106 (24)
> 34	17 (50)	17 (50)
Gravidity		
≤ 2	349 (79.1)	92 (20.9)
3–4	41 (60.3)	27 (39.7)
≥ 5	15 (48.4)	16 (51.6)

Attending ANC* have ANC follow-up

*ANC* antenatal care, *PIH* pregnancy induced hypertension, *APH* antepartum hemorrhage

** Percentage is calculated from row total

The occurrence of still birth was higher in weight < 2.5 kg of newborn compared with weight of greater than 2.5 kg which accounts 62.9 and 22.4% respectively.

### Determinants of still birth

The result of binary logistic regression analysis on independent variables in relation to still birth showed that, age of the women, ANC visit, gravidity, obstructed labour, antenatal risks (placenta previa, abruption placenta, and APH), labour abnormality, length of labor, and the weight of child were found to be significantly associated with still birth. However the result of multiple logistic regression analysis showed that attending ANC, length of labor, uterine rupture, antenatal risks, and weight of the new born were found to be determinants with still birth as clearly depicted in table two.

Women who were attending ANC while they were pregnant were 40% less likely at risk for still birth than those who did not attend (AOR = 0.6, 95% CI 0.39, 0.94). And those who had labor length greater than 24 h were 2.4 times more at risk to have still birth than those who had labor length less than 24 h (AOR = 2.44, 95% CI 1.4, 4.26).

Mothers who developed uterine rupture were 5 times more likely to have still birth than their counterparts (AOR = 4.9, 95% CI 1.67, 14.35). In addition women who have different antenatal risk (placenta previa, abruption placenta) were 4.5 times more likely to have birth outcome of still birth (AOR = 4.58, 95% CI 1.45, 14.48) when compared with their counter parts. This study also showed that weight of baby greater than or equal 2.5 kg were 73% less likely to still birth when compared to those who have less than 2.5 kg (AOR = 0.27, 95% CI 0.14, 0.53) (Table 2).

**Table 2:** Bivariate and multivariable logistic regression analysis of factors associated with still birth in Mizan-Tepi university teaching and Bonga hospitals town (n = 540), May 2016

Variable	Still birth		COR, 95% CI	AOR, 95% CI
	No (%**)	Yes (%**)		
Attending ANC				
No	12 7 (65.8)	66 (34.2)	1.00	1.00
Yes	278 (80.1)	69 (19.9)	0.48 (0.32, 0.71)*	0.6 (0.39, 0.94)*
Length of labor (h)				
< 24	355 (78.7)	96 (21.3)	1.00	1.00
≥ 24	50 (58.2)	39 (43.8)	2.88 (1.79, 4.64)*	2.44 (1.4, 4.26)*
Post-term pregnancy				
No	397 (74.9)	133 (25.1)	1.00	1.00
Yes	8 (80)	2 (20)	0.75 (0.16, 3.56)	0.83 (0.13, 5.19)
Presence of HIV/AIDS				
No	396 (74.7)	134 (25.3)	1.00	1.00
Yes	9 (90)	1 (10)	0.33 (0.04, 2.62)	0.43 (0.02, 10.15)
Anemia				
No	398 (75.2)	131 (24.8)	1.00	1.00
Yes	7 (63.6)	4 (36.4)	1.74 (0.5, 6.02)	0.72 (0.05, 11.48)
Multiple gestation				
No	392 (75.4)	128 (24.6)	1.00	1.00
Yes	13 (65)	7 (35)	1.65 (0.64, 4.22)	0.41 (0.1, 1.68)
Uterine rupture				
No	397 (77.1)	118 (22.9)	1.00	1.00
Yes	8 (32)	17 (68)	7.15 (3.01, 16.98)*	4.9 (1.67, 14.35)*
Presence of co-morbidity				
No	387 (75.2)	128 (24.8)	1.00	1.00
Yes	18 (72)	7 (28)	1.18 (0.48, 2.88)	1.1 (0.1, 11.82)
PIH				
No	388 (75)	129 (25)	1.00	1.00
Yes	17 (73.9)	6 (26.1)	1.06 (0.41, 2.95)	0.84 (0.27, 2.59)
Antenatal risks (APH placenta previa and abruption placenta)				
No	368 (78)	103 (22)	1.00	1.00
Yes	37 (53.6)	32 (46.4)	3.09 (1.83, 5.2)*	4.58 (1.45, 14.48)*
OL				
No	383 (76.8)	116 (23.2)	1.00	1.00
Yes	22 (53.7)	19 (46.3)	2.85 (1.49, 5.45)*	1.4 (0.59, 3.28)
Weight of the new born (kg)				
< 2.5	13 (37.1)	22 (62.9)	1.00	1.00
≥ 2.5	392 (77.6)	113 (22.4)	0.17 (0.08, 0.35)*	0.27 (0.14, 0.53)*
Age (years)				
< 20	50 (80.6)	12 (19.4)	0.24 (0.1, 0.6)*	1.68 (0.59, 4.81)
20–34	338 (76)	106 (24)	0.31 (0.15, 0.64)*	1.6 (0.72, 3.57)
> 34	17 (50)	17 (50)	1.00	1.00
Gravidity				
≤ 2	349 (79.1)	92 (20.9)	0.25 (0.12, 0.52)*	0.51 (0.23, 1.14)
3–4	41 (60.3)	27 (39.7)	0.62 (0.26, 1.45)	0.96 (0.4, 2.27)
≥ 5	15 (48.4)	16 (51.6)	1.00	1.00

*ANC* antenatal care, *PIH* pregnancy induced hypertension

* Shows variable that are significantly associated

** Percentage is calculated from row total

### Discussion

This research finding showed that women who were attending ANC were 40% less likely to have still birth than those who did not attend (AOR = 0.6, 95% CI 0.39, 0.94). The finding of this study is in line with the study done inside and outside of the country that showed women who do not have ANC checkups were at higher risk of stillbirth when compared to their counterparts [12, 13] and study finding in Uganda showed that those who don’t have ANC were risk of still birth [11].

This is indicates the fact that ANC helps the mothers to be screened for certain risk factors, so as to take appropriate measure if risks are detected. Or it creates a good opportunity for health care providers to manage immediately or to establish a future care plan. Moreover, they can provide counseling to mothers and families what should be done to minimize the risks.

In this study the presence of antenatal and intranatal risks (like placenta previa, abruption placenta, other causes of APH) were significantly associated with still birth (AOR = 4.58, 95% CI 1.45, 14.48). Similarly women who have uterine rupture were an important cause of still birth in this study (AOR = 4.9, 95% CI 1.67, 14.35). This result is in agreement with study finding of Karachi, Pakistan, which strengthen presence of antenatal and intranatal risk and uterine rupture as an important cause to still birth [14, 15] and finding of study which are conducted in Hawassa showed as antepartum hemorrhage was determinate factor of still birth [12].

It also showed that women who had labor length greater than 24 h were 2.4 times more at risk to have still birth than labor less than 24 h (AOR = 2.44, 95% CI 1.4, 4.26). This finding is in agreement with the study findings of Bangladesh and Mozambique which showed that prolonged labor were significant factor for still birth [15, 16].. But the study that was done in Uganda showed that the length of labor was not significantly associated with stillbirths [11]. This discrepancy might be due to the difference of study setting and the number of the study population. When the length of labour greater than 24 h the fetus might be exposed for stressful uterine contraction which finally leads to death of the fetus.

The current finding shows that birth weight of baby have significant factor with still birth, means weight of baby ≥ 2.5 kg were less likely to still birth compared than those who have less than 2.5 kg weight (AOR = 0.27, 95% CI 0.14, 0.53). This finding is similar with study finding Hawassa, Karachi Pakistan and Nepal, which shows very low and low birth weight newborns had higher risk of still birth than normal weight newborns [12–14].

### Conclusion and recommendation

Attending ANC, length of labor; uterine rupture, antenatal risks, and weight of the new born were found to be determinant of still birth. The health office of the Bench-Maji and Keffa zones should work hard to improve awareness of the community about attending antenatal care during pregnancy and health professionals should investigate antenatal risk during ANC follow up.

## Limitation of study

- Since this is secondary data it has limitation related with missing of information.
- The study is institution based study that can’t be generalized for the still birth occurred at community level.

## Abbreviations

ANC: antenatal care
AOR: adjusted odds ratio
APH: antepartum hemorrhage
CI: confidence interval
HIV/AIDs: human immunodeficiency virus/acquire immune deficiency syndrome
OL: obstructed labour
OR: odds ratio
PIH: pregnancy induced hypertensive
PNC: postnatal care
PPS: probability proportional to size
SNNPR: Southern Nations, Nationalities, and Peoples Region
WHO: World Health Organization
